# Trends in Prescription Analgesic Use Among Adults With Musculoskeletal Conditions in the United States, 1999-2016

**DOI:** 10.1001/jamanetworkopen.2019.17228

**Published:** 2019-12-11

**Authors:** Andrew Stokes, Kaitlyn M. Berry, Katherine Hempstead, Dielle J. Lundberg, Tuhina Neogi

**Affiliations:** 1 Department of Global Health, Boston University School of Public Health, Boston, Massachusetts; 2 Division of Epidemiology and Community Health, University of Minnesota School of Public Health, Minneapolis; 3 Robert Wood Johnson Foundation, Princeton, New Jersey; 4 Department of Medicine, Boston University School of Medicine, Boston, Massachusetts; 5 Department of Epidemiology, Boston University School of Public Health, Boston, Massachusetts

## Abstract

**Importance:**

Monitoring trends in prescription analgesic use among adults with musculoskeletal conditions provides insight into how changing prescribing practices, guidelines, and policy measures may affect those who need pain management.

**Objective:**

To evaluate trends in prescription opioid use and nonopioid analgesic use among adults with functional limitations attributable to musculoskeletal conditions.

**Design, Setting, and Participants:**

This repeated cross-sectional study uses data from the National Health and Nutrition Examination Study from 1999 to 2016. Data were analyzed from January to July 2019. The participants were adults aged 30 to 79 years who reported functional limitations due to back or neck problems and/or arthritis or rheumatism.

**Main Outcomes and Measures:**

Any use of a prescription opioid or exclusive use of a prescription nonopioid analgesic.

**Results:**

In this population of 7256 adults with 1 or more functional limitations attributable to a musculoskeletal condition (4226 women [59.9%]; 3508 [74.4%] non-Hispanic white individuals; median [interquartile range] age, 63 [53-70] years), opioid use and exclusive nonopioid analgesic use exhibited approximately reciprocal patterns of change from 1999 to 2016. Opioid use increased significantly (difference in prevalence for 2015-2016 vs 1999-2000, 7.2%; 95% CI, 1.3% to 13%; *P* for trend = .002), and exclusive use of nonopioid analgesics decreased significantly (difference in prevalence for 2015-2016 vs 1999-2000, −13%; 95% CI, −19% to −7.5%; *P* for trend < .001) during this period. The increase in any opioid use was driven by long-term rather than short-term use. A crossover in the prevalence of opioid use and exclusive use of nonopioid analgesics occurred between 2003 and 2006, after which opioid use was more prevalent. Between 2013 and 2016, decreases in opioid use were observed among men (difference in prevalence for 2015-2016 vs 2013-2014, −11%; 95% CI, −21% to 1.8%) and participants with less than a high school education (difference, −15%; 95% CI, −24% to −6.1%). During this same period, exclusive nonopioid analgesic use also decreased markedly across the population (difference, −5.3%; 95% CI, −9.1% to −1.5%).

**Conclusions and Relevance:**

The substitution of opioids for nonopioid analgesics between 2003 and 2006 may have occurred as evidence emerged on the cardiovascular risks associated with nonopioid analgesics. Reductions in opioid use between 2013 and 2016 were most substantial among those with low socioeconomic status, who may encounter barriers in accessing alternatives. Despite those decreases, opioid use remained more prevalent in 2015 to 2016 than in 1999 to 2000, suggesting a potentially long tail for the opioid epidemic.

## Key Points

**Question:**

How did prescription analgesic use change among US adults with a potential need for pain management for musculoskeletal conditions from 1999 to 2016?

**Findings:**

In this repeated cross-sectional analysis of nationally representative data from 7256 adults, opioid and nonopioid analgesic use exhibited reciprocal trends, with decreases in nonopioid analgesic use offset by increases in opioid use.

**Meaning:**

Substitution of opioids for nonopioid analgesics may have occurred as evidence emerged on the cardiovascular risks associated with nonopioid analgesics, and despite recent decreases, opioid use remained more prevalent in 2016 than in 1999.

## Teaser

This cross-sectional study uses data from the National Health and Nutrition Examination Study to evaluate trends in prescription opioid use and nonopioid analgesic use from 1999 to 2016 among US adults with functional limitations attributable to musculoskeletal conditions.

## Introduction

Musculoskeletal conditions are a major source of persistent pain and functional limitation and are associated with substantial health care spending in the United States.^1,2^ Musculoskeletal conditions are the leading cause of years lived with disability^3^ and were the third leading cause of disability-adjusted life-years after cardiovascular disease and cancer in the United States in 2016.^3^

In the first decade of the 21st century, opioid therapy for musculoskeletal pain and other chronic noncancer pain conditions expanded rapidly.^4^ Concurrent with these shifts, aggressive marketing of opioids to health care professionals increased the opioid supply available to patients.^5^ As a result, the prevalence of opioid use increased rapidly between 1999 and 2010, peaking at 782 morphine milligram equivalents per capita in 2010 before decreasing to 640 morphine milligram equivalents per capita in 2015 and 513 morphine milligram equivalents per capita in 2017.^6,7^ Despite recent decreases, the morphine milligram equivalents per capita prescribed in 2015 remained approximately triple 1999 levels,^6,8^ and prescription opioids were responsible for more than 17 000 of the more than 42 000 deaths associated with opioid overdose in the United States in 2016.^9^

Although prescribing trends have been described in aggregate, it is less clear how prescription analgesic use patterns have evolved in individuals living with functional limitations attributable to musculoskeletal conditions. Monitoring such trends provides insights into how changing prescribing practices, guidelines, and policy measures may affect those who need long-term pain management. Although a growing body of evidence indicates that long-term opioid use has limited efficacy for musculoskeletal pain^10^ and that nonopioid treatments, including self-care strategies and nonpharmacologic interventions, improve function and health,^11^ pharmacologic therapy including opioid use remains prevalent,^12^ and barriers to nonpharmacologic interventions may delay their adoption.^13^

In the present study, we assessed national trends in prescription pain management among people with functional limitations attributable to musculoskeletal conditions, drawing on data from the National Health and Nutrition Examination Survey (NHANES) from 1999 to 2016. We evaluated trends in the use of prescription opioids overall and separately for short-term and long-term use. We also examined trends in the use of nonopioid analgesics and investigated how trends vary across sociodemographic and health care characteristics.

## Methods

### Study Sample

Fielded continuously since 1999, the NHANES is a nationally representative survey of the noninstitutionalized US population.^14^ The cross-sectional survey consists of a questionnaire, physical examination, and laboratory components and includes detailed information on demographic characteristics, medical history, and prescription medications. Because our analysis relied on publicly available, deidentified data, institutional review board approval was not required (45 CFR §46.102(f)). We followed the Strengthening the Reporting of Observational Studies in Epidemiology (STROBE) reporting guideline.^15^

We included adult participants aged 30 to 79 years with functional limitations attributable to musculoskeletal conditions using data from the nine 2-year survey waves collected between 1999 and 2016. Those who reported a recent cancer diagnosis or were missing data on covariates were excluded from the analysis (eFigure 1 in the Supplement).

### Measures

We obtained data on age, sex (female or male), race (non-Hispanic white, non-Hispanic black, Hispanic, and non-Hispanic other), education (less than high school, high school or equivalent, some college, and college or higher), employment status (not employed or employed), insurance type (none, public only, or any private), and smoking status (never, former, or current) from the NHANES questionnaire. Body mass index (calculated as the weight in kilograms divided by height in meters squared) was categorized as underweight (<18.5) normal weight (≥18.5 to <25), overweight (≥25 to <30), obese I (≥30 to <35), and obese II to III (≥35).

### Musculoskeletal Conditions With Functional Limitation

Because pain questions were not asked routinely in the NHANES, we constructed a sample with potential musculoskeletal pain based on respondents reporting 1 or more functional limitations due to a musculoskeletal condition. Although not a direct measure of pain, functional limitations and pain are closely associated in the context of underlying musculoskeletal conditions.^16,17,18,19,20,21,22,23^

Participants were asked to report whether they had difficulties doing certain activities because of any long-term physical, mental, or emotional health problem or illness, excluding pregnancy. We considered participants to have a functional difficulty if they reported having some difficulty, much difficulty, or were unable to do any of 17 activities (eTable 1 in the Supplement). The maximum level of difficulty was assessed on the basis of individuals’ responses across functional activities.

Participants who reported difficulty with any of the functional activities were then asked about the conditions or health problems that caused them. Our analysis was restricted to participants who reported a functional difficulty due to musculoskeletal conditions, including back or neck problems and arthritis or rheumatism.^24^

### Prescription Pain Management

Participants were also asked about prescription medications taken in the 30 days before the survey. The medication name was recorded from the medication container (83.6%), pharmacy receipt (14.6%), or on the basis of the participant’s verbal response (1.8%). Medications were coded using the Cerner Multum Lexicon Plus and were classified using the Multum Lexicon Therapeutic Classification Scheme, a 3-level nested category system that assigns a therapeutic classification to each drug and each ingredient of the drug.

Participants reporting use of an opioid analgesic (Multum Lexicon Classification: 57 central nervous system agents [level 1], 58 analgesics [level 2], 60 narcotic analgesics, or 191 narcotic analgesic combinations [level 3]) were considered prescription opioid users. Those reporting opioids often used in the treatment of opioid dependence or withdrawal (methadone, buprenorphine, or naloxone) or whose only reported indication for use was opioid dependence or withdrawal were not classified as prescription opioid users.^25^ Opioid use was further classified into exclusive opioid use vs dual use of an opioid and a nonopioid analgesic. Separately, opioid use was also classified into short-term use (<90 days) and long-term use (≥90 days).

Participants reporting use of a nonopioid analgesic (Multum Lexicon Classification: 57 central nervous system agents [level 1], 58 analgesics [level 2], any category other than 60 narcotic analgesic or 191 narcotic analgesic combination [level 3]) but no opioid use were considered exclusive nonopioid analgesic users. Participants reporting any use of opioids or nonopioid analgesics were considered to have prescription analgesic use. A complete list of the generic names for drugs in each category can be found in eTable 2 in the Supplement.

### Statistical Analysis

Data were analyzed from January to July 2019. Using the full NHANES sample from 1999 to 2016 of 7256 participants, we assessed trends in the prevalence of use of prescription opioids and nonopioid analgesics between 1999 and 2016. Estimates were standardized to the overall sample-weighted age distribution using 5-year age intervals. To quantify the changes, we reported the difference in prevalence between 1999 to 2000 and 2015 to 2016 and the *P* value for the overall trend using a logistic regression adjusting for age and a 2-sided Wald test. Because a preliminary analysis showed substantive changes in prescription analgesic use between 2003 to 2004 and 2005 to 2006 and large decreases between 2013 to 2014 and 2015 to 2016, we also estimated differences in prevalence across those periods.

Focusing on any prescription opioid use, we examined trends by age, sex, race/ethnicity, education, employment status, and insurance type. eTable 3 and eTable 4 in the Supplement also present trends in the prevalence of exclusive nonopioid analgesic use and no prescription analgesic use by population subgroup.

We investigated factors associated with prescription pain management using multinomial logistic regression with a 3-level outcome (no prescription analgesic use, any opioid use, and exclusive nonopioid analgesic use), where no prescription analgesic use was considered the referent group. Potential factors included age, sex, race/ethnicity, education, body mass index category, insurance status, smoking status, and survey year. Models were also adjusted for cause of the pain or functional difficulty and maximum level of difficulty reported.

Stata statistical software version 15 (StataCorp) was used for all analyses, and analyses were sample-weighted using NHANES examination weights. We combined the sample weights for each survey cycle (NHANES 1999-2000 to 2015-2016) according to NHANES analytic guidelines so that estimates were representative of the US civilian noninstitutionalized population during an average year of the combined survey period.^26^ We estimated variances using Taylor series linearization with the SVY routine in Stata version 15.

## Results

Pooling across survey waves, the total sample included 7256 adults with 1 or more functional limitations attributable to a musculoskeletal condition. The sample was 59.9% female (4226 participants) and 74.4% non-Hispanic white (3508 participants), and the median (interquartile range) age was 63 (53-70) years (Table 1). A majority of the sample attributed their functional limitations to only back or neck problems (2410 [34.7%]) or only arthritis or rheumatism (2696 [35.5%]), and the remainder (2150 [29.8%]) listed a combination of both conditions as the source of their functional limitations. Respondents’ maximum reported difficulty across activities varied, with 2941 (43.7%) reporting a maximum of some difficulty, 2109 (30.0%) reporting much difficulty, and 2206 (26.4%) reporting they were unable to do activities. eFigure 2 in the Supplement shows the individual functional limitations reported by adults by underlying condition; difficulty stooping, crouching, or kneeling was the most commonly reported limitation across musculoskeletal conditions.

**Table 1.** Characteristics of Adults With Musculoskeletal Conditions With Functional Limitation, National Health and Nutrition Examination Survey, 1999 to 2016^a^

Characteristic	Participants, No. (%) (N = 7256)^b^
Age, median (interquartile range), y	63 (53-70)
Sex	
Female	4226 (59.9)
Male	3030 (40.1)
Race/ethnicity	
Non-Hispanic white	3508 (74.4)
Non-Hispanic black	1580 (11.0)
Hispanic	1753 (9.3)
Non-Hispanic other	415 (5.4)
Education	
Less than high school diploma	2642 (24.7)
High school or equivalent	1795 (27.2)
Some college	1887 (30.7)
Bachelor’s degree or higher	932 (17.4)
Employment	
Not employed	5426 (68.2)
Employed	1830 (31.8)
Insurance type	
None	949 (11.3)
Public only	3267 (36.3)
Any private	3040 (52.4)
Smoking status	
Never	3061 (41.4)
Former	2376 (33.0)
Current	1819 (25.6)
Body mass index category^c^	
Underweight	88 (1.3)
Normal	1338 (19.4)
Overweight	2200 (29.8)
Obese I	1746 (24.2)
Obese II-III	1884 (25.2)
Cause of difficulty^d^	
Back or neck problems	2410 (34.7)
Arthritis or rheumatism	2696 (35.5)
Both back or neck and arthritis or rheumatism	2150 (29.8)
Highest difficulty^e^	
Some difficulty	2941 (43.7)
Much difficulty	2109 (30.0)
Unable to do	2206 (26.4)
Years	
1999-2000	601 (7.6)
2001-2002	640 (9.7)
2003-2004	834 (12.8)
2005-2006	639 (10.0)
2007-2008	946 (10.5)
2009-2010	984 (10.9)
2011-2012	760 (10.8)
2013-2014	857 (12.1)
2015-2016	995 (15.7)

^a^ Statistics are reported for the sample over 9 waves of data that each span 2 years (1999-2016).

^b^ Proportions were weighted using National Health and Nutrition Examination Survey sample weights to be nationally representative.

^c^ Body mass index was calculated as the weight in kilograms divided by height in meters squared.

^d^ Participants who reported difficulty with any functional activities were then asked about the conditions or health problems that cause them to have difficulty or need help and were given the option to report up to 5 health problems. Participants were considered to have musculoskeletal conditions if they chose back or neck problem and/or arthritis or rheumatism as a health condition that caused their functional limitation.

^e^ “Highest difficulty” corresponds to the maximum difficulty reported by each participant according to whether they responded unable to do, had much difficulty with, or had some difficulty with any of the functional activities.

**Figure 1.**
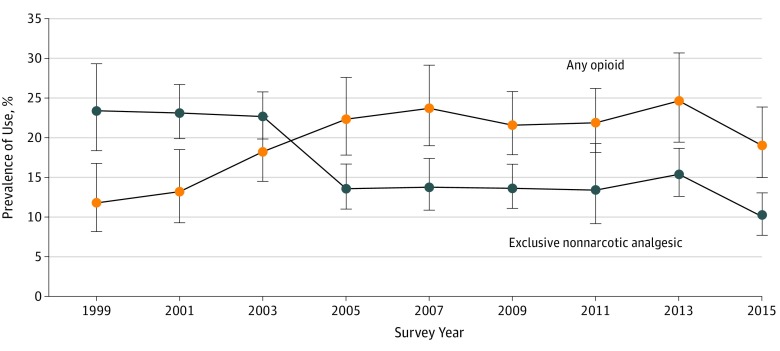
Prevalence of Prescription Opioid and Nonopioid Analgesic Use, 1999 to 2016 Prescription opioid and nonopioid analgesic use exhibited approximately reciprocal patterns of change over the 18-year study period, comparing 1999 to 2000 with 2015 to 2016. Error bars denote 95% CIs.

Prescription opioid and nonopioid analgesic use exhibited approximately reciprocal patterns of change over the 18-year study period, comparing 1999 to 2000 with 2015 to 2016 (Figure 1). Although there was no significant change in the overall use of prescription analgesic medications, any use of opioids increased significantly (difference in prevalence for 2015-2016 vs 1999-2000, 7.2%; 95% CI, 1.3% to 13%; *P* for trend = .002), and exclusive use of nonopioid analgesics decreased significantly (difference in prevalence for 2015-2016 vs 1999-2000, −13%; 95% CI, −19% to −7.5%; *P* for trend < .001) (Table 2).

**Table 2.** Trends in Prescription Analgesic Use Among Adults With Musculoskeletal Conditions With Functional Limitation, National Health and Nutrition Examination Survey, 1999 to 2016

Variable	Prevalence of Use, % (95% CI) (N = 7256)^a^										Difference in Prevalence, % (95% CI)^a^		
	1999-2000 (n = 601)	2001-2002 (n = 640)	2003-2004 (n = 834)	2005-2006 (n = 639)	2007-2008 (n = 946)	2009-2010 (n = 984)	2011-2012 (n = 760)	2013-2014 (n = 857)	2015-2016 (n = 995)	*P* for Trend^b^	2015-2016 vs 1999-2000^c^	2005-2006 vs 2003-2004^d^	2015-2016 vs 2013-2014^e^
Any prescription analgesic^f^	35 (29 to 42)	36 (30 to 43)	41 (36 to 46)	36 (31 to 41)	37 (32 to 43)	35 (31 to 40)	35 (29 to 42)	40 (33 to 47)	29 (24 to 35)	.11	−6.1 (−14 to 2.1)	−5 (−12 to 2.2)	−11 (−20 to −2.2)
Any opioid use^g^	12 (8.2 to 17)	13 (9.3 to 18)	18 (15 to 23)	22 (18 to 28)	24 (19 to 29)	22 (18 to 26)	22 (18 to 26)	25 (19 to 31)	19 (15 to 24)	.002	7.2 (1.3 to 13)	4.1 (−2.0 to 10)	−5.6 (−12 to 1.2)
Short-term (<90 d)	4 (1.5 to 10)^h^	2.5 (1.2 to 5.3)^h^	4.5 (3.3 to 6.1)	4.3 (2.6 to 7)	4.1 (2.9 to 5.7)	4.3 (3 to 6.3)	4.5 (3.1 to 6.6)	2.7 (1.4 to 5.1)^h^	2.7 (1.6 to 4.4)	.50	−1.3 (−5.1 to 2.6)	−0.2 (−2.7 to 2.2)	−0.02 (−2.1 to 2.1)
Long-term (≥90 d)	7.9 (5 to 12)	11 (6.8 to 16)	14 (11 to 18)	18 (15 to 22)	20 (15 to 26)	17 (13 to 22)	17 (14 to 22)	22 (17 to 27)	16 (13 to 21)	<.001	8.5 (3.5 to 13)	4.3 (−0.7 to 9.3)	−5.6 (−12 to 0.6)
Exclusive nonopioid analgesic use^i^	23 (18 to 29)	23 (20 to 27)	23 (20 to 26)	14 (11 to 17)	14 (11 to 17)	14 (11 to 17)	13 (9.1 to 19)	15 (13 to 19)	10 (7.7 to 13)	<.001	−13 (−19 to −7.5)	−9.1 (−13 to −5.2)	−5.3 (−9.1 to −1.5)

^a^ The values for percentage (95% CI) are weighted using National Health and Nutrition Examination Survey sample weights to be nationally representative and standardized to the overall sample-weighted age distribution. Prevalence values of 10% and greater are rounded to the nearest whole number.

^b^ *P* values for trend from 1999 to 2016 are age adjusted.

^c^ Indicates the absolute increase or decrease in prevalence of use between 1999 to 2000 and 2015 to 2016.

^d^ Indicates the absolute increase or decrease in prevalence of use between 2003 to 2004 and 2005 to 2006.

^e^ Indicates the absolute increase or decrease in prevalence of use between 2013 to 2014 and 2015 to 2016.

^f^ Any prescription analgesic refers to any reported use of an opioid analgesic or a nonopioid analgesic in the previous 30 days.

^g^ Any opioid analgesic refers to opioid analgesic use alone or in combination with a nonopioid analgesic.

^h^ The standard error is greater than 30% of the prevalence, suggesting data should be interpreted with caution.

^i^ Exclusively nonopioid analgesic refers to nonopioid analgesic use alone (users of both opioid and nonopioid analgesics are classified into the any opioid use group).

In 1999 to 2000, exclusive use of prescription nonopioid analgesics (prevalence, 23%; 95% CI, 18% to 29%) exceeded use of prescription opioids (prevalence, 12%; 95% CI, 8.2% to 17%). However, after a crossover in the prevalence values between 2003 to 2004 and 2005 to 2006, any use of opioids (prevalence, 22%; 95% CI, 18% to 28%) exceeded exclusive use of nonopioid analgesics (prevalence, 14%; 95% CI, 11% to 17%). Any use of opioids and use of nonopioid analgesics subsequently stabilized and then decreased between 2013 to 2014 and 2015 to 2016, resulting in a marked decrease in the use of any prescription analgesic (difference in prevalence for 2015-2016 vs 2013-2014, −11.0%; 95% CI, −20% to −2.2%). In 2015 to 2016, use of opioids (prevalence, 19%; 95% CI, 15% to 24%) remained more prevalent than exclusive use of nonopioid analgesics (prevalence, 10%; 95% CI, 7.7% to 13%). In addition, despite the decrease in opioid use between 2013 to 2014 and 2015 to 2016, the prevalence of any opioid use remained higher in 2015 to 2016 than in 1999 to 2000 (Table 2).

**Figure 2.**
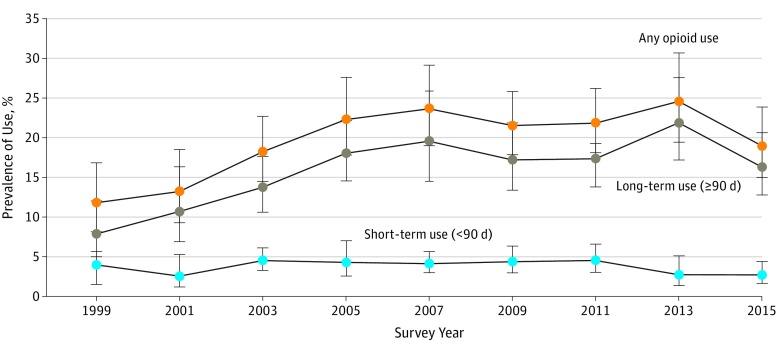
Prevalence of Long-term, Short-term, and Any Opioid Use, 1999 to 2016 The trends in opioid use between 1999 to 2000 and 2015 to 2016 reflected long-term rather than short-term use of opioids. Error bars denote 95% CIs.

The trend in opioid use between 1999 to 2000 and 2015 to 2016 reflected long-term rather than short-term use of opioids (Figure 2), which was less prevalent and remained approximately constant over time. In addition, the overall opioid use trend appeared to be driven by exclusive opioid use rather than opioid use in combination with nonopioid analgesics (eFigure 3 in the Supplement). Differentiating the trends by underlying musculoskeletal conditions, the apparent substitution of nonopioid analgesics with prescription opioids in earlier years was most evident for adults with both back or neck problems and arthritis or rheumatism (eFigure 4 in the Supplement).

The prevalence of any prescription opioid use increased markedly between 1999 to 2000 and 2015 to 2016 for adults older than 50 years (difference, 50-59 years, 16% [95% CI, 1.6% to 30%]; 60-69 years, 10% [95% CI, 0.7% to 19%]; 70-79 years, 8.4% [95% CI, 1% to 16%]), men (difference, 6.8%; 95% CI, 1.1% to 12.5%), women (difference, 9.4%; 95% CI, 0.6% to 18%), Hispanic individuals (difference, 13%; 95% CI, 7.7% to 19%), those who were not employed (difference, 9.7%; 95% CI, 2.5% to 17%), and those with any private insurance (difference, 7.8%; 95% CI, 0.7% to 15%) (Table 3). Between 2013 to 2014 and 2015 to 2016, decreases in opioid use were observed among men (difference, −11%; 95% CI, −21% to 1.8%) and adults with less than a high school education (difference, −15%; 95% CI, −24% to −6.1%). There was an overall decrease in exclusive use of nonopioid analgesics between 2013 to 2014 and 2015 to 2016 (difference, −5.3%; 95% CI, −9.1% to −1.5%) (eTable 3 in the Supplement), creating higher proportions of adults who did not report any prescription analgesic use (eTable 4 in the Supplement).

**Table 3.** Trends in Prescription Opioid Use Among Adults With Musculoskeletal Conditions With Functional Limitation by Population Subgroup, National Health and Nutrition Examination Survey, 1999 to 2016^a^

Variable	Prevalence of Use, % (95% CI) (N = 7256)^b^										Difference in Prevalence, % (95% CI)^b^		
	1999-2000 (n = 601)	2001-2002 (n = 640)	2003-2004 (n = 834)	2005-2006 (n = 639)	2007-2008 (n = 946)	2009-2010 (n = 984)	2011-2012 (n = 760)	2013-2014 (n = 857)	2015-2016 (n = 995)	*P* for Trend^c^	2015-2016 vs 1999-2000^d^	2005-2006 vs 2003-2004^e^	2015-2016 vs 2013-2014^f^
Overall	12 (8.2 to 17)	13 (9.3 to 18)	18 (15 to 23)	22 (18 to 28)	24 (19 to 29)	22 (18 to 26)	22 (18 to 26)	25 (19 to 31)	19 (15 to 24)	.002	7.2 (1.3 to 13)	4.1 (−2.0 to 10)	−5.6 (−12 to 1.2)
Age group, y													
30-49	25 (14 to 41)	18 (12 to 26)	18 (11 to 29)	31 (21 to 43)	29 (21 to 40)	32 (24 to 41)	35 (19 to 56)	27 (19 to 38)	16 (9.4 to 25)	.38	−9 (−24 to 6.2)	13 (−0.7 to 26)	−12 (−23 to 0.1)
50-59	14 (5.2 to 32)^g^	18 (7.3 to 37)^g^	22 (14 to 34)	26 (18 to 36)	32 (21 to 45)	38 (26 to 51)	24 (16 to 35)	33 (19 to 51)	30 (22 to 39)	.02	16 (1.6 to 30)	3.5 (−9.7 to 17)	−3.3 (−21 to 14)
60-69	6.8 (3.6 to 12)	10 (5.5 to 19)	18 (12 to 25)	19 (14 to 25)	23 (18 to 30)	12 (8.5 to 18)	17 (11 to 25)	20 (14 to 28)	16 (9.6 to 27)	.09	9.6 (0.7 to 19)	1.2 (−6.5 to 8.9)	−3.6 (−14 to 6.7)
70-79	7 (3.8 to 13)	9.2 (4.9 to 17)	14 (9.8 to 21)	15 (8.1 to 25)	11 (6.4 to 18)	10 (5.9 to 18)	12 (6.6 to 22)	20 (13 to 29)	15 (10 to 23)	.02	8.4 (1 to 16)	0.2 (−9.3 to 9.7)	−4.3 (−14 to 5.9)
Sex													
Female	14 (8.5 to 23)	15 (11 to 22)	18 (13 to 25)	23 (17 to 30)	24 (19 to 29)	24 (19 to 29)	21 (15 to 27)	24 (19 to 30)	24 (19 to 30)	.01	9.4 (0.6 to 18)	4.7 (−3.8 to 13)	−0.1 (−7.6 to 7.4)
Male	7.9 (5 to 12)	10 (5.1 to 19)^g^	18 (13 to 25)	22 (18 to 26)	23 (17 to 31)	19 (15 to 25)	23 (18 to 29)	26 (18 to 36)	15 (10 to 20)	.02	6.8 (1.1 to 12.5)	3.8 (−2.8 to 10)	−11 (−21 to −1.8)
Race/ethnicity													
Non-Hispanic white	13 (7.7 to 21)	13 (8.2 to 20)	19 (15 to 23)	23 (17 to 30)	25 (19 to 32)	24 (18 to 30)	24 (19 to 30)	26 (20 to 33)	20 (15 to 25)	.003	6.7 (−1.2 to 15)	4.2 (−3.2 to 12)	−6.2 (−14 to 2)
Non-Hispanic black	13 (7.3 to 21)	15 (9 to 24)	18 (12 to 26)	25 (17 to 36)	19 (14 to 26)	18 (13 to 24)	25 (20 to 31)	21 (15 to 28)	18 (14 to 24)	.20	5.7 (−2.4 to 14)	7.4 (−4 to 19)	−2.7 (−11 to 5.4)
Hispanic	5.7 (3.5 to 9)	12 (6.5 to 21)	15 (7.6 to 27)	14 (9.1 to 20)	19 (11 to 30)	19 (15 to 24)	16 (9.4 to 25)	17 (12 to 23)	19 (14 to 25)	.03	13 (7.7 to 19)	−1.1 (−11 to 9.1)	2.2 (−4.9 to 9.3)
Non-Hispanic other	15 (7.9 to 26)	12 (0.8 to 42)^g^	4.8 (1.1 to 19)^g^	14 (7.6 to 24)	19 (10 to 32)	23 (15 to 32)	13 (9.1 to 19)	29 (17 to 45)	13 (6.2 to 25)^g^	.84	−1.9 (−14 to 10)	9.2 (−1 to 19)	−16 (−33 to 0.5)
Education													
Less than high school	11 (6.8 to 19)	12 (6.9 to 20)	24 (19 to 29)	22 (13 to 33)	26 (19 to 35)	22 (17 to 29)	25 (18 to 34)	30 (22 to 39)	14 (11 to 19)	.007	2.9 (−4.0 to 9.9)	−1.8 (−13 to 9)	−15 (−24 to −6.1)
High school or equivalent	14 (8.8 to 22)	18 (11 to 27)	21 (15 to 29)	23 (16 to 33)	23 (15 to 35)	22 (16 to 29)	23 (16 to 31)	25 (14 to 40)	24 (14 to 36)	.13	9.3 (−3.1 to 22)	2.4 (−8.5 to 13)	−1.1 (−18 to 15)
Some college	11 (6.1 to 19)	12 (6.8 to 20)	18 (12 to 25)	24 (16 to 35)	26 (21 to 31)	28 (23 to 34)	24 (18 to 32)	26 (21 to 32)	20 (13 to 28)	.01	8.8 (−0.5 to 18)	6.6 (−4.7 to 18)	−6.4 (−15 to 2.4)
Bachelor’s degree or higher	5.5 (1.4 to 19)^g^	10 (4.4 to 21)^g^	10 (6.4 to 16)	11 (7.5 to 15)	21 (15 to 30)	9.6 (4.3 to 20)^g^	15 (8.6 to 24)	13 (8.8 to 20)	14 (8.9 to 22)	.25	8.6 (−0.6 to 18)	0.5 (−5.5 to 6.4)	0.8 (−7.1 to 8.7)
Employment													
Not employed	13 (9.8 to 18)	16 (12 to 21)	23 (19 to 29)	29 (23 to 37)	29 (24 to 34)	26 (21 to 31)	24 (20 to 30)	29 (22 to 37)	23 (17 to 30)	.003	9.7 (2.5 to 17)	5.9 (−2.3 to 14)	−5.7 (−15 to 3.6)
Employed	6.2 (3 to 13)^g^	8.7 (4.2 to 17)^g^	14 (10 to 19)	13 (10 to 17)	15 (9.4 to 24)	11 (7.5 to 16)	14 (8.2 to 24)	13 (8.2 to 20)	10 (5.6 to 17)	.15	3.8 (−3.1 to 11)	−1.1 (−6.3 to 4.1)	−3 (−11 to 4.7)
Insurance type													
None	13 (7.1 to 23)	7.7 (2.5 to 21)^g^	8.9 (4 to 19)^g^	14 (8.4 to 22)	9.5 (6.2 to 14)	17 (9.6 to 28)	14 (6.9 to 25)^g^	12 (5.4 to 23)^g^	22 (12 to 37)	.98	8.6 (−5.7 to 23)	5 (−4.2 to 14)	10.2 (−4.3 to 25)
Public only	15 (12 to 20)	15 (9.9 to 21)	28 (20 to 37)	31 (25 to 38)	30 (26 to 36)	27 (19 to 36)	26 (20 to 32)	31 (24 to 39)	21 (15 to 29)	.16	5.8 (−1.8 to 13)	3.7 (−6.3 to 14)	−9.9 (−20 to 0.02)
Any private	8.7 (5.5 to 14)	14 (8.6 to 22)	16 (12 to 20)	20 (14 to 28)	21 (16 to 27)	23 (19 to 27)	24 (16 to 34)	22 (17 to 28)	17 (11 to 24)	.007	7.8 (0.7 to 15)	4.3 (−3.3 to 12)	−5.5 (−13 to 2.3)

^a^ Prescription opioid use refers to opioid analgesic use alone or in combination with a nonopioid analgesic.

^b^ The values for percentage (95% CI) are weighted using National Health and Nutrition Examination Survey sample weights to be nationally representative and standardized to the overall sample-weighted age distribution. Prevalence values of 10% and greater are rounded to the nearest whole number.

^c^ *P* values for trend from 1999 to 2016 are age adjusted.

^d^ Indicates the absolute increase or decrease in prevalence of use between 1999 to 2000 and 2015 to 2016.

^e^ Indicates the absolute increase or decrease in prevalence of use between 2003 to 2004 and 2005 to 2006.

^f^ Indicates the absolute increase or decrease in prevalence of use between 2013 to 2014 and 2015 to 2016.

^g^ The standard error is greater than 30% of the prevalence, suggesting data should be interpreted with caution.

Across the entire 1999 to 2016 period, non-Hispanic black adults had 0.79 times the odds (95% CI, 0.65-0.96) of reporting any prescription opioid use compared with non-Hispanic white adults (eTable 5 in the Supplement). Reduced odds of opioid use were also found for employed adults (vs not employed). Elevated odds of opioid use were seen for some college education (vs less than high school), public or private insurance (vs no insurance), current and former smoking (vs never), attributing functional limitations to multiple musculoskeletal conditions (vs back or neck problems only), and reporting higher levels of difficulty with functional tasks (vs some difficulty). Adults with public or private insurance (vs none), those with arthritis or rheumatism or multiple musculoskeletal conditions (vs back or neck problems only), and those with higher levels of difficulty also had higher odds of exclusive prescription nonopioid analgesic use.

## Discussion

In the present study, we documented trends in prescription analgesic use among US adults with functional limitations attributable to musculoskeletal conditions. Although overall trends in prescription analgesic use have been previously investigated, few studies have examined trends in individuals with musculoskeletal conditions using national data from a contemporary period, despite the fact that such individuals are highly affected by chronic pain and are, therefore, likely to be directly affected by changes in prescribing practices, policies, and guidelines.

The study had several key findings. First, we observed a marked decrease in the use of nonopioid analgesics among adults with musculoskeletal conditions and a coincident acceleration in the use of prescription opioids between 2003 to 2004 and 2005 to 2006. Although identifying the causes of this trend are beyond the scope of the present study, the decrease in nonopioid analgesic use may be associated with the withdrawal of the cyclooxygenase-2 inhibitor rofecoxib from the market after the release of data indicating that its use was associated with myocardial infarction and stroke.^27^ The use of nonsteroidal anti-inflammatory drugs also decreased, likely reflecting the overall cardiovascular class effect concerns for both nonselective and selective nonsteroidal anti-inflammatory drugs alike, with a similar substitution effect between nonopioid and opioid analgesics over this period. Notably, trends in both classes of medications appeared to plateau after the shifts between 2003 to 2004 and 2005 to 2006, suggesting that the changes over this period were not simply a data anomaly.

We found evidence that opioid use remained more prevalent in 2015 to 2016 than 1999 to 2000, suggesting a long tail to the opioid epidemic in this population. The extent to which trends in prescription opioid use reflect increases in the prevalence or severity of pain vs a greater reliance on opioids for treating existing pain is unclear. However, the increase in the prevalence of opioid use documented in this sample was more dramatic than the corresponding rate of change in the national population,^28^ suggesting that the second mechanism—expansion of opioid use in chronic pain management—contributed to the overall trend in opioid use at the population level. It is unlikely that these trends represented increases in the prevalence of musculoskeletal pain and/or pain severity because the trends persisted after adjusting for the severity of functional limitations. Furthermore, the prevalence of painful conditions has not increased so markedly over this time period.^29^ The observation that expanded access to opioids is an important factor in the overall increase in opioid use is consistent with a large body of evidence implicating supply-side factors as causes of the opioid epidemic, including shifts in clinician norms toward pain treatment, changes in national guidelines, the World Health Organization’s analgesic ladder, and aggressive marketing of opioids.^5,30,31,32^

Our findings included decreases in prescription opioid use between 2013 to 2014 and 2015 to 2016. Notably, reductions were also observed for nonopioid analgesics, leading to an overall 11% absolute decrease in the percentage of individuals receiving prescription pain management between 2013 to 2014 and 2015 to 2016. This recent decrease may reflect changes in physician prescribing practices as awareness of the opioid epidemic grew and as Centers for Disease Control and Prevention guidelines about reducing opioid use were developed, made public for comment, and ultimately released in early 2016.^33,34^ Decreases in use could also reflect reductions in demand for prescription analgesics by patients.

The fact that the present study was restricted to patients with potential needs for pain management also raises the concerning possibility that an unmet need for pain management has increased over this period.^35^ Such a trend would be alarming given evidence that untreated chronic pain may prompt patients with chronic pain to seek out illicit heroin or fentanyl.^36^ In addition, several recent studies^37,38^ based on data from the National Violent Death Reporting System have found a high rate of chronic pain among suicide decedents, and recent research and commentary^12,39,40,41^ on opioid discontinuation have suggested that recent increases in the suicide death rate may be linked to changes in pain treatment. Our findings also indicated that the subpopulations most affected by decreases in prescription analgesic use included those with less than high school education, who may have reduced means for seeking out alternative forms of pain management, including nonpharmacologic services such as physical therapy. Barriers are known to exist for these services, including costs, access, insurance coverage, ability to take time off work, and patient skepticism,^13^ so greater public health planning is needed to ensure that alternatives to opioids are available and accessible to all patients.

### Limitations

This study has several limitations. First, because self-reported pain data were not routinely available, we studied a population with functional limitations due to musculoskeletal conditions as a potential proxy for musculoskeletal pain. Although pain and activity limitations are closely associated and musculoskeletal conditions are typically painful,^15,20,22^ it is possible that some adults in the sample were not presently experiencing pain. Second, we were not able to adjust for severity of pain, but we were able to approximate pain severity using data on severity of functional limitations. Third, the NHANES did not routinely collect data on over-the-counter medications, and thus our study is limited to examining prescription pain medication. This may have led to an underestimation of the proportion of individuals receiving nonopioid analgesics in our study. Fourth, we did not investigate the use of nonanalgesic medications for the treatment of pain in this study because of the lack of specific data on these drugs or nonpharmacologic interventions in the NHANES. Additional studies incorporating these approaches are critical to obtaining a complete picture of trends in pain management in the population.

## Conclusions

We found a dramatic decrease in the use of nonopioid analgesics from 2003 to 2006 that may have occurred as evidence emerged on the cardiovascular risks associated with nonopioid analgesics. We also identified large decreases in use of any prescription analgesic between 2013 and 2016 that were particularly evident among those with less than high school education. In addition, despite recent decreases, an overall increase in opioid use still occurred between 1999 and 2016, with the trends largely explained by expansion of long-term opioid use. Future studies are needed to examine the implications of the current study on potential unmet need for pain management and the extent to which decreases in prescription pain management are being counterbalanced by use of other pain management strategies, such as physical therapy and other nonpharmacologic services outside the medical system. Changes in patterns of pain management are likely to affect the health of adults with musculoskeletal pain, which also warrants further research.

## Notes

Supplement. **eTable 1.** Functional Tasks Included in the Analyses, NHANES 1999-2016 **eTable 2.** Pain Management Prescriptions Included in the Analysis by Category, NHANES 1999-2016 **eTable 3.** Trends in Exclusive Use of Prescription Nonopioid Analgesics Among Adults With Musculoskeletal Conditions With Functional Limitation by Population Subgroup, NHANES, 1999-2016 **eTable 4.** Trends in No Prescription Pain Management Among Adults With Musculoskeletal Conditions With Functional Limitation by Population Subgroup, NHANES, 1999-2016 **eTable 5.** Predictors of Prescription Pain Analgesic Use Among Adults With Musculoskeletal Conditions With Functional Limitation, NHANES 1999-2016 **eFigure 1.** Flowchart of Study Inclusion/Exclusion Criteria **eFigure 2.** Functional Difficulties by Reported Cause, NHANES 1999-2016 **eFigure 3.** Trends in Prescription Analgesic Use Among Adults With Musculoskeletal Conditions With Functional Limitation, NHANES 1999-2016 **eFigure 4.** Trends in Prescription Analgesic Use Among Adults With Functional Limitations Due to Either Musculoskeletal Condition, Back or Neck Problems Only, Arthritis or Rheumatism Only, and Both Back/Neck Problems and Arthritis/Rheumatism, NHANES 1999-2016
