# Supplementary material for: Trends in Prescription Analgesic Use Among Adults With Musculoskeletal Conditions in the United States, 1999-2016
Source: JAMA Netw Open. 2019 Dec 11;2(12):e1917228. doi: 10.1001/jamanetworkopen.2019.17228 (PMC6991204; doi:10.1001/jamanetworkopen.2019.17228)
Supplement: Supplement. — eTable 1. Functional Tasks Included in the Analyses, NHANES 1999-2016 eTable 2. Pain Management Prescriptions Included in the Analysis by Category, NHANES 1999-2016 eTable 3. Trends in Exclusive Use of Prescription Nonopioid Analgesics Among Adults With Musculoskeletal Conditions With Functional Limitation by Population Subgroup, NHANES, 1999-2016 eTable 4. Trends in No Prescription Pain Management Among Adults With Musculoskeletal Conditions With Functional Limitation by Population Subgroup, NHANES, 1999-2016 eTable 5. Predictors of Prescription Pain Analgesic Use Among Adults With Musculoskeletal Conditions With Functional Limitation, NHANES 1999-2016 eFigure 1. Flowchart of Study Inclusion/Exclusion Criteria eFigure 2. Functional Difficulties by Reported Cause, NHANES 1999-2016 eFigure 3. Trends in Prescription Analgesic Use Among Adults With Musculoskeletal Conditions With Functional Limitation, NHANES 1999-2016 eFigure 4. Trends in Prescription Analgesic Use Among Adults With Functional Limitations Due to Either Musculoskeletal Condition, Back or Neck Problems Only, Arthritis or Rheumatism Only, and Both Back/Neck Problems and Arthritis/Rheumatism, NHANES 1999-2016 [file jamanetwopen-2-e1917228-s001.pdf]

Supplementary Online Content

Stokes A, Berry KM, Hempstead K, Lundberg DJ, Neogi T. Trends in prescription analgesic use among adults with musculoskeletal conditions in the United States, 1999-2016. *JAMA Netw Open*. 2019;2(12):e1917228. doi:10.1001/jamanetworkopen.2019.17228

eTable 1. Functional Tasks Included in the Analyses, NHANES 1999-2016

eTable 2. Pain Management Prescriptions Included in the Analysis by Category, NHANES 1999-2016

eTable 3. Trends in Exclusive Use of Prescription Nonopioid Analgesics Among Adults With Musculoskeletal Conditions With Functional Limitation by Population Subgroup, NHANES, 1999-2016

eTable 4. Trends in No Prescription Pain Management Among Adults With Musculoskeletal Conditions With Functional Limitation by Population Subgroup, NHANES, 1999-2016

eTable 5. Predictors of Prescription Pain Analgesic Use Among Adults With Musculoskeletal Conditions With Functional Limitation, NHANES 1999-2016

eFigure 1. Flowchart of Study Inclusion/Exclusion Criteria

eFigure 2. Functional Difficulties by Reported Cause, NHANES 1999-2016

eFigure 3. Trends in Prescription Analgesic Use Among Adults With Musculoskeletal Conditions With Functional Limitation, NHANES 1999-2016

eFigure 4. Trends in Prescription Analgesic Use Among Adults With Functional Limitations Due to Either Musculoskeletal Condition, Back or Neck Problems Only, Arthritis or Rheumatism Only, and Both Back/Neck Problems and Arthritis/Rheumatism, NHANES 1999-2016

This supplementary material has been provided by the authors to give readers additional information about their work.

© 2019 Stokes A et al. *JAMA Network Open.*

eTable 1. Functional Tasks Included in the Analyses, NHANES 1999-2016

The next questions ask about difficulties you may have doing certain activities because of a health problem. By "health problem" we mean any long-term physical, mental, or emotional problem or illness, not including pregnancy.

By yourself, how much difficulty do you have…

**Task Label**	**Full Question**
Stooping, crouching, kneeling	… stooping, crouching, or kneeling?
Lifting or carrying	… lifting or carrying something as heavy as 10 pounds, like a sack of potatoes or rice?
Doing chores	… doing chores around the house, like vacuuming, sweeping, dusting, or straightening up?
Preparing meals	… preparing your own meals?
Walking between rooms	… walking from one room to another on the same level?
Standing from armless chair	… standing up from an armless straight chair?
Getting in and out of bed	… getting in or out of bed?
Using fork, knife, cup	… eating, like holding a fork, cutting food, or drinking from a glass?
Dressing yourself	… dressing yourself, including tying shoes, working zippers, and doing buttons?
Standing for long periods	… standing or being on your feet for about two hours?
Sitting for long periods	… sitting for about two hours?
Reaching over head	… reaching up over your head?
Grasp/holding small objects	… using your fingers to grasp or handle small objects?
Going out to movies, events	… going out to things like shopping, movies, or sporting events?
Attending social events	… participating in social activities, like visiting friends, attending clubs or meetings, or going to parties?
Relaxing at home	… doing things to relax at home or for leisure, like reading, watching TV, sewing, listening to music?

© 2019 Stokes A et al. *JAMA Network Open.*

eTable 2. Pain Management Prescriptions Included in the Analysis by Category, NHANES 1999-2016[a]

**Opioid Analgesics**
acetaminophen; butalbital; caffeine; codeine
acetaminophen; codeine
acetaminophen; hydrocodone
acetaminophen; oxycodone
acetaminophen; propoxyphene
acetaminophen; tramadol
aspirin; butalbital; caffeine; codeine
aspirin; oxycodone
chlorpheniramine; hydrocodone
chlorpheniramine; hydrocodone; phenylephrine
codeine
codeine; guaifenesin
codeine; guaifenesin; pseudoephedrine
codeine; promethazine
fentanyl
guaifenesin; hydrocodone
hydrocodone
hydrocodone; ibuprofen
hydrocodone; phenylpropanolamine
hydromorphone
meperidine
morphine
naloxone; pentazocine
oxycodone
oxymorphone
propoxyphene
tapentadol
tramadol
**Nonopioid Analgesics**
aceclofenac
acetaminophen; butalbital
acetaminophen; butalbital; caffeine
acetaminophen; caffeine; isometheptene mucate
acetaminophen; dextromethorphan; pseudoephedrine
acetaminophen; dichloralphenazone; isometheptene
acetaminophen; phenyltoloxamine
analgesics - unspecified
aspirin
aspirin; butalbital; caffeine
aspirin; caffeine; orphenadrine
aspirin; carisoprodol
aspirin; dipyridamole
belladonna; ergotamine; phenobarbital
betamethasone; indomethacin; methocarbamol
caffeine; ergotamine
celecoxib
choline salicylate; magnesium salicylate
diclofenac
diclofenac; misoprostol
diflunisal

© 2019 Stokes A et al. *JAMA Network Open.*

eletriptan
etodolac
fenoprofen
flurbiprofen
frovatriptan
ibuprofen
indomethacin
ketoprofen
ketorolac
meloxicam
nabumetone
naproxen
naratriptan
nimesulide
oxaprozin
piroxicam
rizatriptan
rofecoxib
salsalate
sulindac
sumatriptan
tolmetin
valdecoxib
zolmitriptan

[a]Medications were coded using the Cerner Multum Lexicon Plus and classified using the Multum Lexicon Therapeutic Classification Scheme, a three-level nested category system that assigns a therapeutic classification to each drug and each ingredient of the drug. Opioids often used in treatment for opioid dependence or withdrawal (methadone, buprenorphine, and buprenorphine; naloxone) were excluded from our "any opioid use" outcome variable. Naloxone; pentazocine was retained due to the combination with pentazocine.

© 2019 Stokes A et al. *JAMA Network Open.*

eTable 3. Trends in Exclusive Use of Prescription Nonopioid Analgesics Among Adults With Musculoskeletal Conditions With Functional Limitation by Population Subgroup, NHANES, 1999-2016 (n=7,256)[a]

	**Prevalence of Use, % (95% CI)[b]**										**Difference in prevalence, % (95% CI)[b]**		
	**1999-2000** n= 601	**2001-2002** n= 640	**2003-2004** n= 834	**2005-2006** n= 639	**2007-2008** n= 946	**2009-2010** n= 984	**2011-2012** n= 760	**2013-2014** n= 857	**2015-2016** n= 995	**P for Trend[c]**	**2015-2016 vs 1999-2000[d]**	**2005-2006 vs 2003-2004[e]**	**2015-2016 vs 2013-2014[f]**
**Overall**	23.4 (18.4-29.3)	23.1 (19.9-26.7)	22.7 (19.8-25.7)	13.6 (11.0-16.7)	13.8 (10.8-17.4)	13.6 (11.1-16.7)	13.4 (9.1-19.3)	15.4 (12.6-18.6)	10.1 (7.7-13.0)	<0.001	-13.3 (-19.1 to -7.5)	-9.1 (-13.0 to -5.2)	-5.3 (-9.1 to -1.5)
**Age group**													
30-49 years	16.7 (8.1-31.5)	16.6 (9.9-26.3)	17.3 (10.0-28.1)	8.7 (5.2-14.2)	13.5 (6.8-25.3)g	8.1 (3.9-16.1)g	9.2 (4.4-18.2)g	16.5 (9.9-26.3)	11.1 (5.9-19.8)	0.17	-5.7 (-18.4 to 7.1)	-8.6 (-18.1 to 1.0)	-5.4 (-15.6 to 4.7)
50-59 years	33.0 (21.7-46.7)	20.9 (15.0-28.5)	31.1 (21.7-42.4)	11.9 (7.4-18.6)	12.6 (7.1-21.5)	16.3 (9.6-26.4)	21.1 (12.2-34.2)	16.7 (9.2-28.3)	8.7 (5.3-14.0)	<0.001	-24.3 (-37.1 to -11.5)	-19.2 (-30.5 to -7.8)	-8.0 (-17.9 to 1.9)
60-69 years	17.1 (12.4-23.2)	29.8 (23.3-37.2)	21.9 (14.6-31.5)	17.3 (12.4-23.5)	15.7 (10.8-22.2)	11.6 (8.1-16.2)	14.0 (8.5-22.3)	16.2 (11.7-22.2)	9.2 (6.2-13.3)	<0.001	-7.9 (-14.1 to -1.8)	-4.6 (-14.3 to 5.1)	-7.1 (-13.1 to -1.0)
70-79 years	28.9 (21.4-37.8)	22.7 (17.0-29.7)	22.0 (14.4-32.1)	14.9 (9.0-23.7)	13.0 (9.3-17.9)	19.2 (14.7-24.7)	8.2 (4.3-15.0)	12.6 (7.7-19.9)	11.5 (7.6-17.0)	<0.001	-17.4 (-26.5 to -8.3)	-7.1 (-18.1 to 3.9)	-1.1 (-8.4 to 6.2)
**Gender**													
Female	24.6 (19.0-31.2)	26.9 (23.0-31.3)	23.6 (19.4-28.3)	14.6 (11.2-18.8)	16.1 (12.1-21.2)	15.4 (11.9-19.7)	14.9 (9.9-21.9)	15.4 (12.1-19.3)	11.3 (8.4-14.9)	<0.001	-13.3 (-19.9 to -6.7)	-9.0 (-14.6 to -3.3)	-4.1 (-8.7 to 0.5)
Male	21.5 (14.3-31.0)	19.1 (14.5-24.7)	20.5 (17.8-23.5)	13.0 (7.6-21.5)	11.2 (8.3-15.0)	11.3 (7.0-17.8)	11.2 (6.6-18.3)	15.0 (10.6-20.7)	8.5 (5.0-13.9)	<0.001	-13.0 (-22.0 to -4.0)	-7.5 (-14.6 to -0.4)	-6.5 (-12.8 to -0.2)
**Race/ethnicity**													
NH White	23.2 (17.6-30.0)	23.4 (19.2-28.3)	22.6 (19.3-26.2)	13.2 (10.2-17.0)	13.3 (9.4-18.4)	11.4 (8.5-15.1)	13.8 (8.0-22.8)	15.7 (11.8-20.5)	8.8 (5.6-13.6)	<0.001	-14.4 (-21.4 to -7.4)	-9.3 (-13.9 to -4.7)	-6.9 (-12.4 to -1.3)
NH Black	20.7 (16.6-25.5)	20.3 (12.0-32.1)	22.4 (17.9-27.5)	17.4 (11.4-25.5)	12.7 (10.4-15.5)	17.7 (11.9-25.5)	15.1 (10.8-20.6)	15.0 (9.9-21.9)	15.2 (9.9-22.5)	0.06	-5.5 (-12.8 to 1.8)	-5.0 (-13.1 to 3.1)	0.2 (-8.0 to 8.5)
Hispanic	25.3 (19.6-32.1)	21.5 (15.2-29.4)	22.8 (15.3-32.6)	12.2 (7.6-19.0)	20.0 (14.6-26.7)	21.6 (17.0-27.0)	10.1 (6.7-14.9)	18.9 (13.4-25.8)	15.4 (11.6-20.2)	0.003	-9.9 (-17.1 to -2.6)	-10.7 (-20.5 to -0.8)	-3.4 (-10.6 to 3.8)
NH Other	45.8 (35.4-56.7)	24.5 (10.9-46.3)g	30.2 (20.5-42.1)	11.3 (2.6-38.0)g	14.9 (5.9-33.1)g	18.9 (12.6-27.2)	14.6 (6.4-29.9)g	9.9 (4.7-19.6)g	9.0 (4.4-17.7)g	<0.001	-36.8 (-48.5 to -25.2)	-18.9 (-36.8 to -1.0)	-0.9 (-10.0 to 8.2)
**Education**													
< High school	22.6 (16.3-30.4)	26.7 (20.6-33.7)	21.6 (15.6-29.1)	12.5 (8.7-17.7)	13.8 (10.9-17.2)	16.6 (10.9-24.3)	12.8 (8.4-19.0)	10.5 (7.1-15.1)	13.2 (9.9-17.5)	<0.001	-9.3 (-17.0 to -1.7)	-9.1 (-16.8 to -1.3)	2.8 (-2.5 to 8.0)
High school	21.3 (14.0-31.0)	21.1 (15.3-28.2)	21.1 (16.6-26.6)	10.8 (5.4-20.2)g	10.3 (5.5-18.4)	15.1 (9.8-22.5)	7.1 (3.7-13.5)g	12.4 (7.0-21.0)	11.3 (7.1-17.6)	0.001	-10.0 (-19.5 to -0.5)	-10.4 (-18.7 to -2.1)	-1.1 (-9.3 to 7.1)
Some college	25.2 (17.4-35.0)	23.4 (15.0-34.5)	25.8 (19.6-33.1)	15.9 (12.7-19.8)	16.3 (10.7-24.1)	12.6 (8.2-19.0)	13.3 (7.8-21.8)	17.6 (11.2-26.6)	8.7 (5.5-13.7)	<0.001	-16.5 (-25.8 to -7.2)	-9.8 (-17.1 to -2.6)	-8.9 (-17.2 to -0.6)
≥ College	26.9 (19.1-36.4)	19.0 (11.6-29.7)	21.1 (15.4-28.3)	15.9 (10.0-24.2)	11.2 (5.5-21.3)g	8.3 (4.6-14.6)	18.4 (11.4-28.5)	22.8 (17.9-28.6)	9.8 (5.8-16.1)	0.014	-17.1 (-26.7 to -7.5)	-5.3 (-14.4 to 3.8)	-13.0 (-20.1 to -5.9)
**Employment**													
Not employed	20.5 (14.1-28.8)	25.7 (20.4-31.7)	22.2 (18.5-26.5)	12.0 (8.5-16.8)	10.6 (8.3-13.3)	13.0 (9.6-17.3)	14.5 (10.3-20.2)	14.0 (11.1-17.4)	11.2 (8.3-15.0)	<0.001	-9.2 (-16.9 to -1.6)	-10.2 (-15.7 to -4.7)	-2.7 (-7.1 to 1.6)
Employed	27.4 (17.3-40.6)	19.8 (13.7-27.8)	21.7 (17.3-26.9)	15.8 (12.0-20.6)	19.5 (14.6-25.6)	16.5 (9.8-26.3)	10.7 (4.9-22.1)g	18.1 (11.7-26.8)	8.6 (4.5-16.0)g	<0.001	-18.8 (-31.3 to -6.4)	-5.8 (-12.0 to 0.3)	-9.4 (-18.4 to -0.5)
**Insurance type**													
None	5.7 (1.7-17.2)g	19.6 (11.5-31.4)	17.4 (7.6-34.9)g	14.7 (11.6-18.3)	8.1 (3.9-16.2)g	17.5 (11.9-25.1)	9.5 (4.6-18.5)g	4.5 (1.8-10.5)g	3.8 (1.5-8.9)g	0.45	-1.9 (-8.9 to 5.1)	-2.7 (-16.0 to 10.6)	-0.7 (-5.6 to 4.2)
Public only	24.2 (15.0-36.6)	31.7 (26.1-37.9)	26.8 (19.7-35.3)	17.9 (10.0-29.9)	14.8 (11.2-19.3)	16.1 (11.1-22.9)	13.8 (8.0-22.8)	16.8 (12.8-21.6)	13.5 (10.3-17.5)	<0.001	-10.7 (-21.6 to 0.2)	-8.9 (-21.0 to 3.1)	-3.2 (-8.6 to 2.2)
Any private	25.0 (20.5-30.1)	21.1 (17.4-25.4)	22.5 (18.6-26.9)	12.8 (10.7-15.3)	15.1 (10.3-21.4)	11.9 (8.6-16.2)	11.5 (5.8-21.5)g	16.1 (12.3-20.9)	7.9 (4.7-13.0)	<0.001	-17.1 (-23.1 to -11.1)	-9.7 (-14.3 to -5.2)	-8.3 (-13.9 to -2.6)

NHANES = National Health and Nutrition Examination Survey, CI = confidence interval, NSAIDs = nonsteroidal anti-inflammatory drugs.

[a] Exclusive use of prescription non-opioid analgesics refers to use of one or more non-opioid analgesics without dual use of a opioid analgesic (dual users are grouped with exclusive opioid users).

[b] The values for % (95% CI) are weighted using NHANES sample weights to be nationally representative and standardized to the overall sample-weighted age distribution.

[c] P values for trend from 1999 to 2016 are age-adjusted.

© 2019 Stokes A et al. *JAMA Network Open.*

[d] Indicates the absolute increase or decrease in prevalence of use between 1999-2000 and 2015-2016.

[e] Indicates the absolute increase or decrease in prevalence of use between 2003-2004 and 2005-2006.

[f] Indicates the absolute increase or decrease in prevalence of use between 2013-2014 and 2015-2016.

[g] The standard error is > 30% of the prevalence, suggesting data should be interpreted with caution.

© 2019 Stokes A et al. *JAMA Network Open.*

eTable 4. Trends in No Prescription Pain Management Among Adults With Musculoskeletal Conditions With Functional Limitation by Population Subgroup, NHANES, 1999-2016 (n=7,256)[a]

	Prevalence of Use, % (95% CI)[b]										Difference in prevalence, % (95% CI)[b]		
	**1999-2000** n= 601	**2001-2002** n= 640	**2003-2004** n= 834	**2005-2006** n= 639	**2007-2008** n= 946	**2009-2010** n= 984	**2011-2012** n= 760	**2013-2014** n= 857	**2015-2016** n= 995	**P for Trend**[c]	**2015-2016 vs 1999-2000**[d]	**2005-2006 vs 2003-2004**[e]	**2015-2016 vs 2013-2014**[f]
**Overall**	64.8 (58.1-71.0)	63.7 (57.2-69.7)	59.1 (53.9-64.1)	64.1 (58.5-69.3)	62.5 (56.8-67.9)	64.8 (60.5-68.9)	64.7 (57.8-71.1)	60.0 (52.6-66.9)	70.9 (65.0-76.2)	0.11	6.1 (-2.1 to 14.3)	5.0 (-2.2 to 12.1)	10.9 (2.2 to 19.7)
**Age group**													
30-49 years	58.4 (42.8-72.6)	65.7 (56.4-74.0)	64.3 (49.9-76.5)	60.1 (45.8-72.9)	57.0 (44.6-68.6)	60.2 (51.4-68.3)	55.8 (36.8-73.3)	56.0 (43.6-67.7)	73.1 (62.8-81.4)	0.84	14.7 (-2.5 to 31.8)	-4.1 (-22.8 to 14.5)	17.1 (2.3 to 31.9)
50-59 years	53.3 (35.5-70.3)	61.3 (45.0-75.4)	46.6 (34.5-59.2)	62.3 (50.2-73.0)	55.5 (40.5-69.6)	45.7 (34.2-57.6)	54.7 (42.5-66.4)	50.3 (35.9-64.7)	61.6 (52.8-69.6)	0.69	8.3 (-10.8 to 27.4)	15.7 (-0.7 to 32.1)	11.3 (-5.1 to 27.6)
60-69 years	76.1 (69.6-81.6)	59.9 (50.3-68.7)	60.4 (50.5-69.5)	63.8 (57.4-69.6)	61.2 (51.3-70.3)	76.1 (69.4-81.6)	69.1 (60.1-76.9)	63.8 (52.3-73.9)	74.4 (62.3-83.7)	0.19	-1.7 (-13.5 to 10.1)	3.4 (-7.6 to 14.3)	10.7 (-4.0 to 25.4)
70-79 years	64.1 (53.1-73.7)	68.1 (60.1-75.1)	63.6 (55.1-71.3)	70.5 (56.6-81.4)	75.9 (67.5-82.8)	70.4 (63.3-76.7)	79.4 (69.8-86.5)	67.8 (61.1-73.8)	73.1 (65.7-79.4)	0.03	9.0 (-2.9 to 21.0)	6.9 (-7.5 to 21.3)	5.3 (-3.6 to 14.3)
**Gender**													
Female	61.1 (54.1-67.6)	57.6 (52.3-62.7)	58.3 (49.9-66.3)	62.6 (56.6-68.2)	60.2 (53.4-66.6)	61.0 (55.0-66.7)	64.6 (55.2-72.9)	60.8 (53.1-67.9)	65.0 (58.4-71.0)	0.12	3.9 (-5.0 to 12.8)	4.3 (-5.4 to 14.0)	4.2 (-5.2 to 13.6)
Male	70.6 (59.5-79.8)	70.8 (61.3-78.7)	61.2 (54.5-67.6)	65.0 (57.0-72.2)	65.5 (58.6-71.8)	69.6 (65.1-73.8)	65.8 (58.0-72.7)	58.9 (48.9-68.2)	76.8 (71.4-81.5)	0.32	6.2 (-4.7 to 17.1)	3.8 (-5.9 to 13.4)	17.9 (7.4 to 28.5)
**Race/ethnicity**													
NH White	64.0 (55.2-71.9)	63.5 (55.0-71.2)	58.7 (52.7-64.4)	63.8 (56.8-70.2)	61.9 (55.2-68.2)	65.0 (58.3-71.0)	62.3 (53.0-70.8)	58.5 (49.7-66.8)	71.6 (64.8-77.6)	0.25	7.6 (-2.5 to 17.8)	5.1 (-3.5 to 13.7)	13.1 (2.8 to 23.4)
NH Black	66.7 (59.5-73.2)	64.8 (53.5-74.6)	59.9 (53.9-65.6)	57.5 (48.2-66.3)	68.0 (59.9-75.1)	64.5 (58.6-70.1)	59.9 (51.6-67.7)	64.1 (55.7-71.6)	66.5 (58.0-74.0)	0.69	-0.2 (-10.4 to 10.0)	-2.4 (-12.8 to 8.1)	2.4 (-8.5 to 13.3)
Hispanic	69.0 (61.7-75.5)	66.7 (52.7-78.2)	62.4 (56.2-68.2)	74.2 (67.5-79.9)	60.9 (50.7-70.2)	59.4 (53.6-64.9)	74.1 (65.7-81.1)	64.5 (54.7-73.2)	65.7 (58.7-72.1)	0.80	-3.3 (-12.6 to 5.9)	11.8 (3.6 to 20.0)	1.2 (-9.8 to 12.2)
NH Other	39.3 (24.1-57.0)	63.8 (44.7-79.4)	65.0 (51.1-76.7)	74.7 (62.0-84.2)	66.1 (46.0-81.7)	58.6 (50.4-66.3)	72.2 (58.7-82.6)	61.1 (48.2-72.6)	78.0 (65.5-86.9)	0.03	38.7 (20.0 to 57.4)	9.7 (-6.4 to 25.9)	17.0 (1.2 to 32.7)
**Education**													
< High school	65.9 (56.5-74.3)	61.5 (56.8-65.9)	54.9 (49.3-60.3)	65.7 (56.3-74.1)	60.0 (51.0-68.4)	61.1 (53.9-67.9)	62.4 (53.1-70.8)	59.9 (52.4-67.0)	72.4 (66.3-77.7)	0.19	6.4 (-3.7 to 16.6)	10.9 (0.8 to 20.9)	12.5 (3.6 to 21.4)
High school	64.4 (51.3-75.7)	61.0 (50.9-70.3)	57.9 (48.9-66.5)	65.9 (53.9-76.1)	66.3 (52.8-77.7)	63.0 (54.1-71.2)	70.2 (60.2-78.6)	62.8 (50.2-74.0)	65.1 (53.0-75.4)	0.43	0.7 (-15.4 to 16.8)	7.9 (-5.8 to 21.7)	2.2 (-13.7 to 18.1)
Some college	63.8 (52.9-73.3)	64.7 (51.0-76.3)	56.6 (47.3-65.5)	59.9 (51.1-68.1)	58.1 (50.6-65.2)	59.1 (52.4-65.6)	62.4 (51.9-71.8)	56.2 (44.8-67.0)	71.5 (61.8-79.5)	0.42	7.7 (-5.4 to 20.8)	3.2 (-8.8 to 15.3)	15.3 (1.5 to 29.1)
≥ College	67.6 (53.1-79.4)	70.9 (61.0-79.2)	68.5 (61.1-75.1)	73.4 (64.5-80.7)	67.4 (57.4-76.0)	82.1 (71.0-89.6)	66.7 (58.9-73.8)	63.9 (56.0-71.2)	76.1 (65.5-84.3)	0.28	8.5 (-7.2 to 24.1)	4.8 (-5.5 to 15.2)	12.2 (0.5 to 23.8)
**Employment**													
Not employed	66.2 (57.2-74.2)	58.1 (50.1-65.7)	54.5 (48.0-60.8)	58.7 (52.3-64.9)	60.9 (56.4-65.3)	61.3 (57.5-65.0)	61.3 (54.3-67.8)	57.3 (49.2-65.1)	65.8 (59.1-71.9)	0.28	-0.4 (-10.7 to 9.8)	4.3 (-4.4 to 12.9)	8.5 (-1.4 to 18.3)
Employed	66.3 (52.7-77.7)	71.5 (61.7-79.5)	64.0 (57.5-70.0)	71.0 (64.4-76.8)	65.3 (55.5-73.9)	72.6 (61.5-81.5)	74.9 (63.6-83.5)	68.9 (58.5-77.7)	81.4 (75.1-86.4)	0.04	15.1 (1.8 to 28.4)	7.0 (-1.5 to 15.4)	12.5 (1.8 to 23.2)
**Insurance type**													
None	81.1 (70.0-88.8)	72.7 (58.4-83.5)	73.7 (55.7-86.2)	71.4 (64.0-77.8)	82.3 (75.1-87.8)	65.5 (57.3-72.9)	77.0 (64.2-86.1)	84.0 (72.4-91.3)	74.4 (58.8-85.6)	0.68	-6.7 (-22.5 to 9.1)	-2.3 (-18.6 to 14.0)	-9.5 (-25.3 to 6.2)
Public only	60.5 (47.7-71.9)	53.7 (46.3-60.8)	45.6 (39.0-52.4)	50.9 (39.6-62.0)	54.8 (49.9-59.6)	57.2 (49.6-64.5)	60.6 (53.1-67.7)	52.2 (45.0-59.4)	65.4 (57.7-72.3)	0.04	4.9 (-8.8 to 18.6)	5.2 (-7.4 to 17.9)	13.1 (3.2 to 23.0)
Any private	66.3 (61.1-71.1)	64.7 (55.1-73.3)	61.5 (54.9-67.8)	66.9 (60.8-72.5)	64.0 (55.6-71.7)	65.4 (60.8-69.8)	64.6 (52.7-75.0)	61.8 (55.1-68.0)	75.6 (67.9-81.9)	0.13	9.3 (1.0 to 17.6)	5.4 (-3.0 to 13.8)	13.8 (4.7 to 23.0)

© 2019 Stokes A et al. *JAMA Network Open.*

NHANES = National Health and Nutrition Examination Survey, CI = confidence interval, NH = non-Hispanic

[a] Participants who reported no prescription opioid or non-opioid analgesic use were considered to have no prescription analgesic pain management.

[b] The values for % (95% CI) are weighted using NHANES sample weights to be nationally representative and standardized to the overall sample-weighted age distribution.

[c] P values for trend from 1999 to 2016 are age-adjusted.

[d] Indicates the absolute increase or decrease in prevalence of use between 1999-2000 and 2015-2016.

[e] Indicates the absolute increase or decrease in prevalence of use between 2003-2004 and 2005-2006.

[f] Indicates the absolute increase or decrease in prevalence of use between 2013-2014 and 2015-2016.

[g] The standard error is > 30% of the prevalence, suggesting data should be interpreted with caution.

© 2019 Stokes A et al. *JAMA Network Open.*

eTable 5. Predictors of Prescription Pain Analgesic Use Among Adults With Musculoskeletal Conditions With Functional Limitation, NHANES 1999-2016 (n=7,256)[a]

	**Any Opioid Use[b]**			**Exclusive Non-Opioid Analgesic Use[c]**		
	**RRR[d]**	**95% CI**	**p-value**	**RRR[d]**	**95% CI**	**p-value**
Age group						
30-49 years	Ref	-	-	Ref	-	-
50-59 years	0.99	0.75-1.29	0.92	1.35	0.97-1.89	0.08
60-69 years	0.55	0.43-0.71	<0.001	0.96	0.71-1.31	0.79
70-79 years	0.36	0.27-0.49	<0.001	0.83	0.59-1.16	0.27
Gender						
Female	Ref	-	-	Ref	-	-
Male	0.89	0.76-1.04	0.15	0.80	0.67-0.96	0.02
Race/ethnicity						
Non-Hispanic White	Ref	-	-	Ref	-	-
Non-Hispanic Black	0.79	0.65-0.96	0.02	1.03	0.84-1.26	0.79
Hispanic	0.69	0.54-0.88	0.003	1.14	0.92-1.43	0.23
Non-Hispanic Other	0.69	0.48-1.00	0.05	1.01	0.69-1.48	0.96
Education						
< High school diploma	Ref	-	-	Ref	-	-
High school or equivalent	1.10	0.87-1.38	0.43	0.89	0.68-1.15	0.37
Some college	1.21	1.00-1.47	0.05	1.17	0.94-1.46	0.17
Bachelor's degree or higher	0.82	0.61-1.09	0.17	1.08	0.84-1.40	0.53
Employment						
Unemployed	Ref	-	-	Ref	-	-
Employed	0.66	0.53-0.83	<0.001	1.12	0.89-1.43	0.33
BMI category						
Underweight	1.96	0.96-4.01	0.07	1.42	0.68-2.97	0.34
Normal	Ref	-	-	Ref	-	-
Overweight	1.13	0.87-1.46	0.35	1.09	0.87-1.37	0.44
Obese I	1.10	0.82-1.48	0.51	1.02	0.80-1.30	0.88
Obese II/III	1.33	1.03-1.73	0.03	1.20	0.89-1.60	0.23
Insurance type						
None	Ref	-	-	Ref	-	-
Public only	2.02	1.49-2.75	<0.001	2.44	1.72-3.48	<0.001
Any private	1.82	1.31-2.54	<0.001	1.85	1.34-2.56	<0.001
Smoking status						

© 2019 Stokes A et al. *JAMA Network Open.*

Never	Ref	-	-	Ref	-	-
Former	1.26	1.01-1.58	0.04	1.12	0.91-1.38	0.28
Current	1.55	1.27-1.90	<0.001	1.01	0.77-1.32	0.93
Cause of difficulty[e]						
Back or neck problems	Ref	-	-	Ref	-	-
Arthritis/rheumatism	0.82	0.65-1.03	0.09	1.73	1.39-2.15	<0.001
Both back/neck and arthritis	1.63	1.35-1.96	<0.001	1.82	1.44-2.32	<0.001
Highest difficulty[f]						
Some difficulty	Ref	-	-	Ref	-	-
Much difficulty	2.10	1.67-2.63	<0.001	1.58	1.27-1.97	<0.001
Unable to do	3.55	2.82-4.46	<0.001	1.90	1.55-2.32	<0.001
Survey year						
1999-2000	Ref	-	-	Ref	-	-
2001-2002	1.04	0.65-1.67	0.86	1.02	0.72-1.44	0.91
2003-2004	1.39	0.87-2.23	0.17	1.06	0.75-1.49	0.74
2005-2006	1.83	1.13-2.97	0.02	0.60	0.42-0.85	0.005
2007-2008	1.83	1.14-2.95	0.01	0.62	0.43-0.89	0.01
2009-2010	1.76	1.12-2.76	0.01	0.62	0.44-0.88	0.008
2011-2012	1.76	1.14-2.71	0.01	0.57	0.34-0.96	0.03
2013-2014	1.91	1.16-3.15	0.01	0.68	0.47-1.00	0.05
2015-2016	1.40	0.88-2.25	0.16	0.37	0.25-0.54	<0.001

NHANES = National Health and Nutrition Examination Survey, CI = confidence interval, RRR = relative risk ratio

[a] We investigated predictors of prescription pain management using multinomial logistic regression using the 3-level outcome (no prescription analgesic use, any opioid use, and exclusive non-opioid analgesic use) where no prescription analgesic use was considered the reference for the outcome.

[b] Any opioid analgesic refers to opioid analgesic use alone or in combination with a non-opioid analgesic

[c] Exclusive non-opioid analgesic use = non-opioid analgesic use alone (users of both opioid and non-opioid analgesics are classified into the opioid group).

[d] All data are weighted to be nationally representative.

[e] Participants who reported difficulty with any functional activities were then asked about the conditions or health problems that cause them to have difficulty or need help and were given the option to report up to five health problems. Our analysis was restricted to participants who reported a functional difficulty due to “arthritis/rheumatism” or “back or neck problem” or both.

[f] “Highest difficulty” corresponds to the maximum difficulty reported by each participant based on whether they responded “unable to do”, “much difficulty”, or “some difficulty” to any of the functional activities.

© 2019 Stokes A et al. *JAMA Network Open.*

eFigure 1. Flowchart of Study Inclusion/Exclusion Criteria

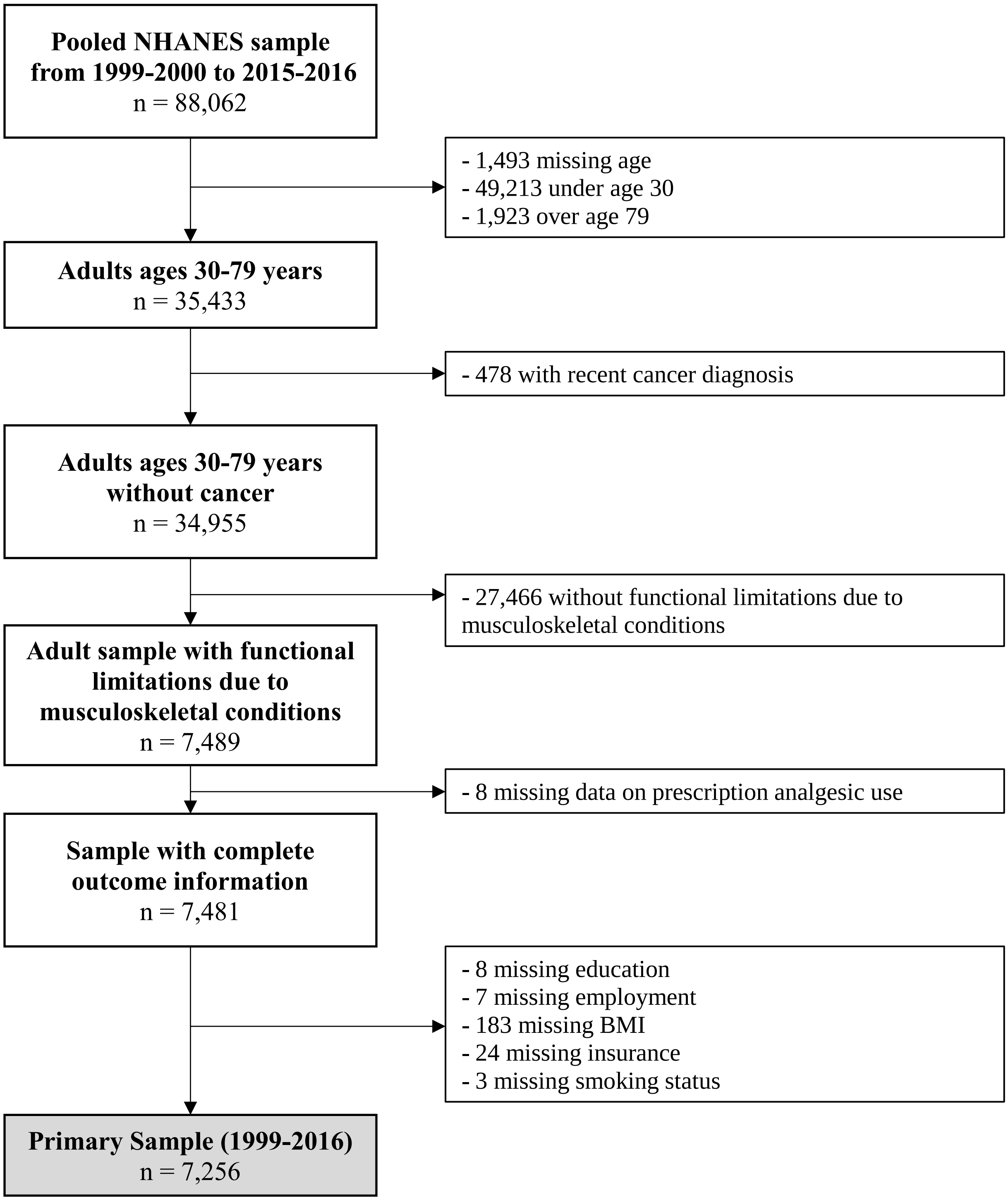

© 2019 Stokes A et al. *JAMA Network Open.*

eFigure 2. Functional Difficulties by Reported Cause, NHANES 1999-2016 (n=7,256)

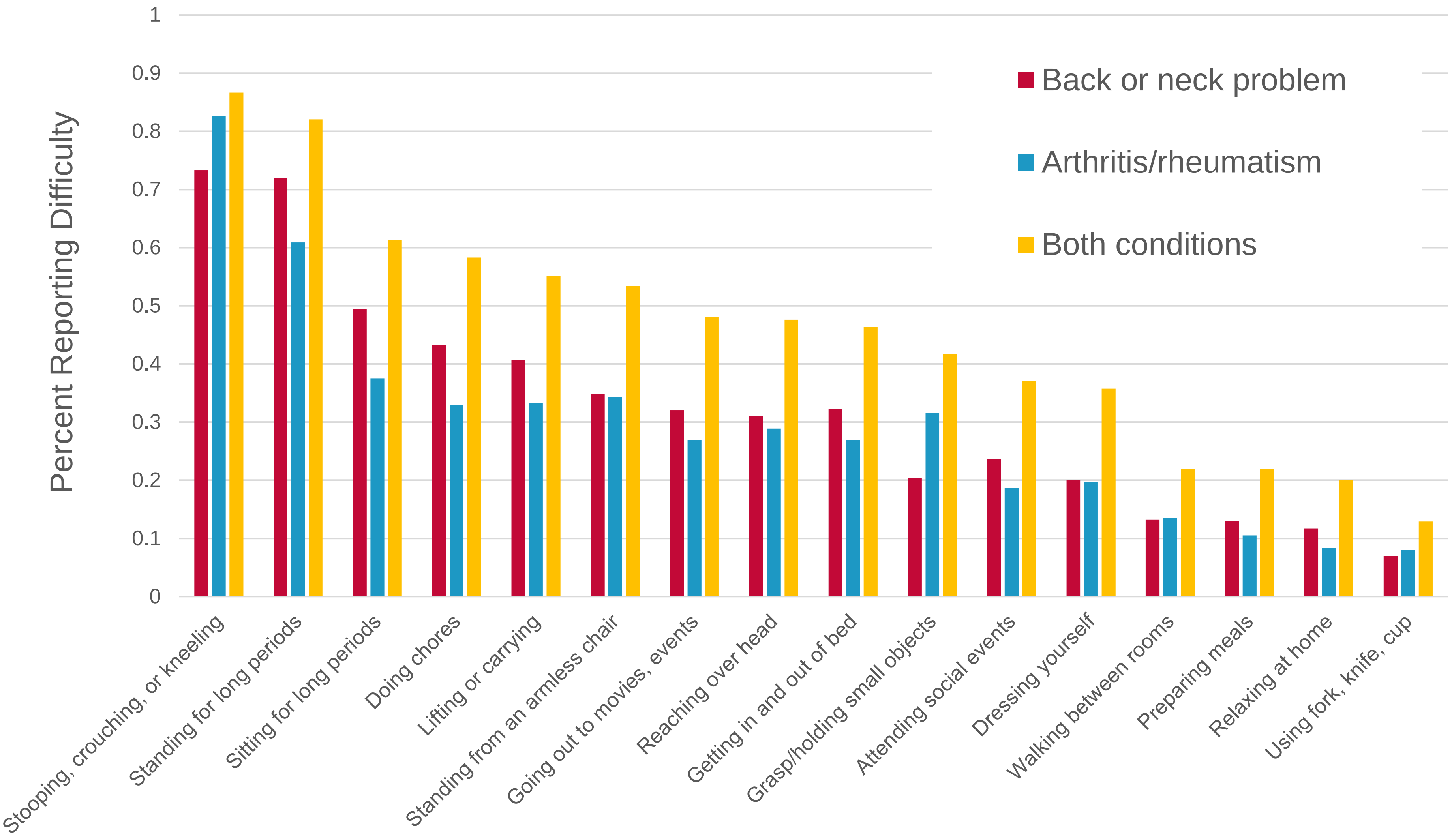

Participants were asked to report whether they had difficulties doing certain activities because of any long-term physical, mental or emotional health problem or illness, excluding pregnancy. We considered participants to have a functional difficulty if they reported having "some difficulty", "much difficulty", or were "unable to do" any of 17 functional activities. Participants who reported difficulty with any of the functional activities were then asked about the conditions or health problems that cause them to have difficulty or need help and were given the option to report up to five health problems. Our analysis was restricted to participants with musculoskeletal pain, defined as those who reported a functional difficulty due to "arthritis/rheumatism" and/or "back or neck problems" as a health condition that caused their functional limitation. All data are weighted to be nationally representative.

© 2019 Stokes A et al. *JAMA Network Open.*

eFigure 3. Trends in Prescription Analgesic Use Among Adults With Musculoskeletal Conditions With Functional Limitation, NHANES 1999-2016 (n=7,256)

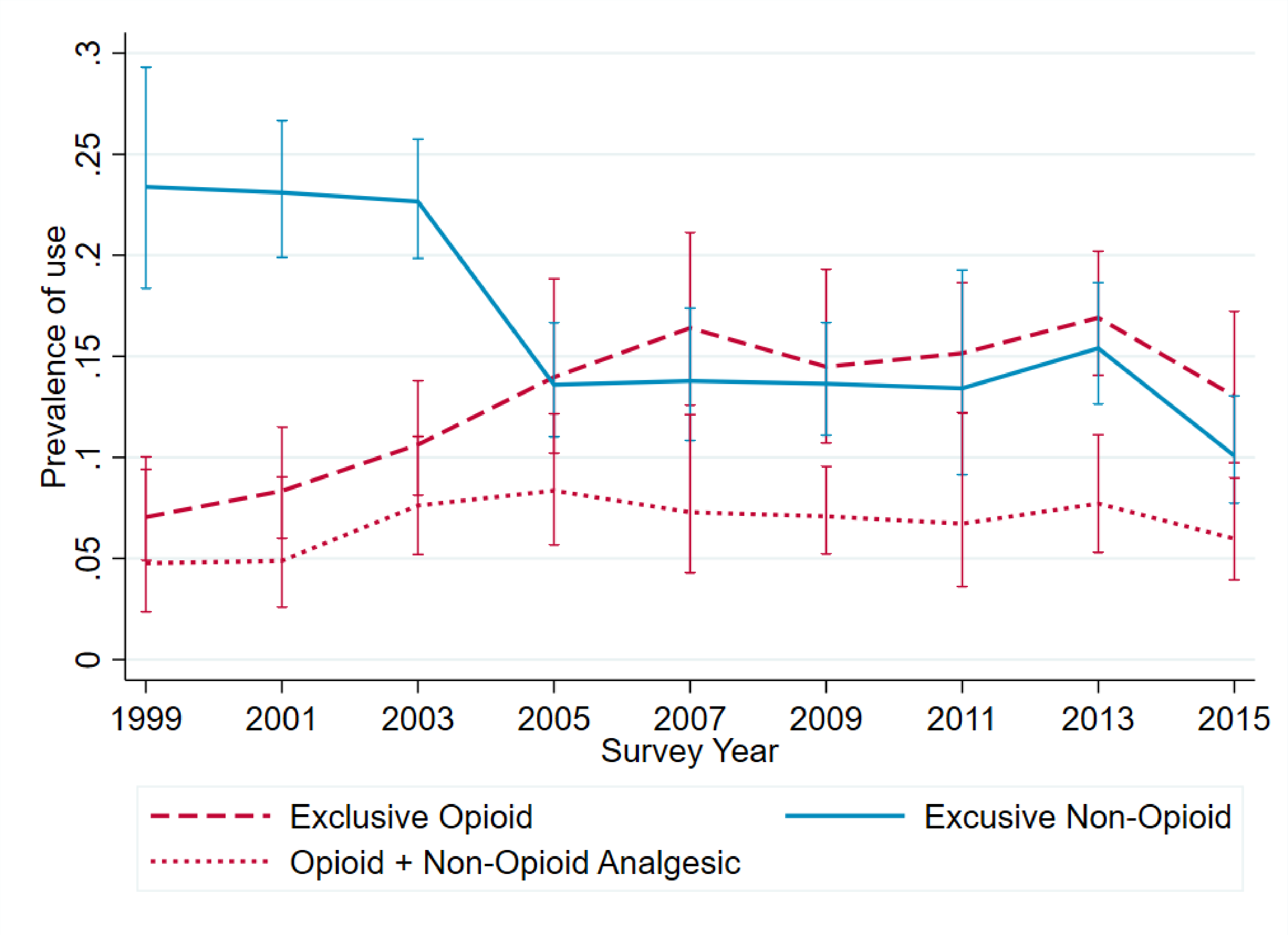

Figure 1 compared the trends in any prescription opioid use (alone or in combination with a non-opioid analgesic) with trends in exclusive use of non-opioid analgesics. e**Figure 3** separates the opioid use category into exclusive opioid use and dual use of an opioid and a non-opioid analgesic. Participants were considered to have musculoskeletal pain if they reported a functional limitation and chose “back or neck problem” and/or “arthritis/rheumatism” as a health condition that caused their functional limitation. All data are weighted to be nationally representative and standardized to the overall sample-weighted age distribution. Error bars indicate 95% CIs.

© 2019 Stokes A et al. *JAMA Network Open.*

eFigure 4. Trends in Prescription Analgesic Use Among Adults With Functional Limitations Due to Either Musculoskeletal Condition (n=7,256), Back or Neck Problems Only (n=2,410), Arthritis or Rheumatism Only (n=2,696), and Both Back/Neck Problems and Arthritis/Rheumatism (n=2,150), NHANES 1999-2016

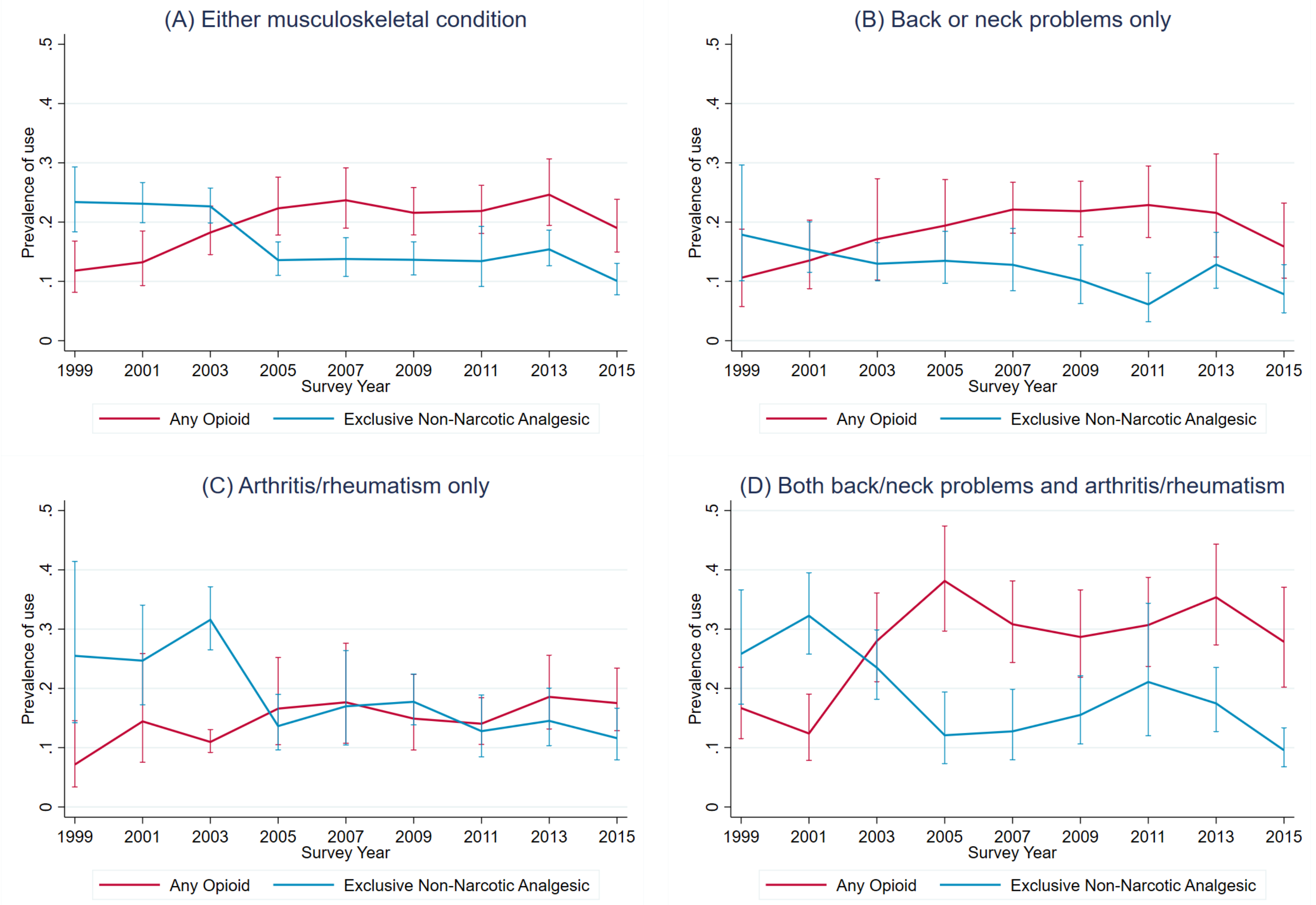

Participants were considered to have musculoskeletal pain if they reported a functional limitation and chose “back or neck problem” and/or “arthritis/rheumatism” as a health condition that caused their functional limitation. Figure S4-A shows the trends for either conditions together (same as Figure 1) while B-D subset the sample into adults with functional limitations due to back or neck problems only; arthritis/rheumatism only; or both musculoskeletal conditions. All data are weighted to be nationally representative and and standardized to the overall sample-weighted age distribution. Error bars indicate 95% CIs.

© 2019 Stokes A et al. *JAMA Network Open.*
